# Addressing future work ability of employees in GP consultations: results of a cross-sectional study

**DOI:** 10.1186/s12995-021-00299-y

**Published:** 2021-03-22

**Authors:** Anna T. Ehmann, Peter Martus, Achim Siegel, Monika A. Rieger

**Affiliations:** 1 Institute of Occupational and Social Medicine and Health Services Research, University Hospital Tübingen, Wilhelmstraße 27, 72074 Tübingen, Germany; 2 Institute for Clinical Epidemiology and Applied Biometry, University Hospital Tübingen, Silcherstr. 5, 72076 Tübingen, Germany

**Keywords:** Perceived work ability, Future work ability, General practice, Cross-sectional survey, Self-administered questionnaire, Germany, Integrated healthcare

## Abstract

**Objectives:**

In this study we examined to what extent members of a best-practice integrated healthcare model in Germany discussed their subjective future work ability with their general practitioner (GP); furthermore, we examined independent variables which explain whether future work ability is discussed.

**Methods:**

In a cross-sectional survey, 1168 (out of 3218 invited) integrated healthcare members responded to a standardized questionnaire. This study includes *n* = 475 employed respondents who were at most 65 years old. We determined the (relative) frequency of employed members up to 65 years who had already discussed their subjective future work ability with their GP. By means of logistic regression analysis, explanatory variables were identified which statistically explained the discussion of future work ability with their GP.

**Results:**

*N* = 80 (16.8%) respondents stated they had discussed their future work ability with their GP. A multiple logistic regression analysis showed the following results: The odds ratio for discussing future work ability is increased the more satisfied respondents are with their general practitioner, the worse they assess their current work ability in relation to the physical demands of the job, and when respondents suffer from one or more chronic diseases (Nagelkerke’s pseudo-R^2^ = 0.13).

**Conclusions:**

Even in this healthcare setting, employees up to the age of 65 rarely discussed their subjective future work ability with their GP. This suggests that the issue ‘future work ability’ is even less commonly discussed in other community-based care settings in Germany. It seems that health care providers involved in acute care only sporadically take this issue into consideration - despite the great importance of maintaining work ability.

**Supplementary Information:**

The online version contains supplementary material available at 10.1186/s12995-021-00299-y.

## Abstract (de)

**Zielsetzung:**

In dieser Studie untersuchten wir, wie häufig Mitglieder eines Best-Practice-Modells der Integrierten Versorgung in Deutschland ihre subjektive künftige Arbeitsfähigkeit mit ihrem Hausarzt (GP) besprechen; darüber hinaus untersuchten wir unabhängige Variablen, die erklären, ob die künftige Arbeitsfähigkeit thematisiert wird.

**Methoden:**

In einer Querschnittserhebung beantworteten 1168 (von 3218 Eingeladenen) Mitglieder der Integrierten Versorgung einen standardisierten Fragebogen. Diese Studie umfasst *n* = 475 berufstätige Befragte, die höchstens 65 Jahre alt waren. Wir ermittelten die Häufigkeit von erwerbstätigen Mitgliedern bis 65 Jahre, die ihre subjektive künftige Arbeitsfähigkeit bereits mit ihrem Hausarzt besprochen hatten. Mit Hilfe einer logistischen Regressionsanalyse wurden Prädiktoren ermittelt, die erklären, ob die künftige Arbeitsfähigkeit mit dem Hausarzt besprochen wird.

**Ergebnisse:**

*N* = 80 (16,8%) der Befragten gaben an, ihre künftige Arbeitsfähigkeit mit ihrem Hausarzt besprochen zu haben. Eine multiple logistische Regressionsanalyse ergab folgende Ergebnisse: Die Odds Ratio für die Besprechung der künftigen Arbeitsfähigkeit ist umso höher, je zufriedener die Befragten mit ihrem Hausarzt sind, je schlechter sie selbst ihre derzeitige Arbeitsfähigkeit in Bezug auf die körperlichen Arbeitsanforderungen einschätzen und wenn die Befragten chronisch krank sind (Nagelkerke-Pseudo-R^2^ = 0,13).

**Schlussfolgerungen:**

Selbst im hier vorliegenden Versorgungskontext besprachen Erwerbstätige bis zum Alter von 65 Jahren ihre künftige Arbeitsfähigkeit nur selten mit ihrem Hausarzt. Dies deutet darauf hin, dass das Thema “künftige Arbeitsfähigkeit” in anderen populationsbezogenen Settings der Gesundheitsversorgung in Deutschland noch seltener besprochen wird. Es scheint, dass in der Akutversorgung tätige Leistungserbringer dieses Thema nur sporadisch berücksichtigen - trotz der großen Bedeutung des Erhalts der Arbeitsfähigkeit.

## Background

Work is an essential aspect of life and can strongly influence the health and well-being of the individual [1–3]. Also diseases can have an impact on one’s work ability [2]. The concept ‘work ability’ has been coined by Ilmarinen et al. since 1980 in the course of practice-oriented research projects in Finland [4]. Work ability is defined as the individuals’ potential to perform their work tasks while taking into account personal health, working conditions as well as mental resources [5, 6]. Work ability thus describes the ability to perform in relation to specific work requirements, in particular in relation to the work tasks to be performed on site [7]; work ability is therefore always characterized by the person and the situation [7, 8]. Both the individual prerequisites of employees and the external demands of work change over time, which requires a distinction between current and future work ability [9]. Future work ability refers to the ability of a person to carry out his or her work under current working conditions in the upcoming years [9]. The prognosis of one’s own work ability two years from now is well known for the prediction of receiving disability pension [10, 11]. Good work ability is related to a high quality of work, high productivity and enjoying remaining in one’s job [12]. Early efforts to maintain work ability throughout working life are deemed the key to a good life in retirement [13].

In patient care, the general practitioner (GP) plays a key role. In addition, the GP may also play a significant role in all questions related to work and health [14], especially as many, particularly small and medium-sized enterprises do not have an occupational physician on a regular basis in Germany [15]. For this reason, employees of small and medium-sized companies are often dependent on the expertise and skills of their GP for work-related questions [16]. Diseases arising from the patient’s work or occupation and the effects of these and general diseases on the patient’s work ability are considered to be important issues in general practice [17]. As an international systematic review revealed, depending on the type of complaint, up to a third of the patients surveyed in general practices had work-related complaints [2]. Although patients’ health problems often have a work-related origin or general diseases interact with work-related demands, the work itself is rarely addressed in consultations with GPs [2]. This has been shown, for example, by a Dutch study that recorded consultations with GPs on video, where only one third of workers with musculoskeletal complaints mentioned their work in the recorded consultations [18]. During those consultations in which the work of the patients was addressed, in 70% of the cases the patients themselves mentioned their work without the GP asking about the patients’ work situation [18].

The awareness of GPs for work-related health problems is often limited [19, 20]. The role of GPs in assessing work ability and in facilitating employees’ return to work may also be limited [21]. This may be due to insufficient knowledge about the patients’ employment and lack of information about working conditions [21]. As research studies in Germany [22–28], Great Britain [17] and the United States [29] concluded, a different set of priorities, limited time for appointments or inexperience in dealing with work-related health problems, e.g. lack of knowledge about patients’ working conditions and possible referral, are regarded as barriers to a work-related consideration of health complaints in general practice. However, there are many good reasons for GPs to integrate work-related aspects more strongly; one important reason is to promote the health and well-being of employees [14]. The awareness of work-related health complaints is of great importance for the individual patient and also from a public health perspective [30].

In Germany, GPs are the main contacts for the provision of primary care and continuous, often life-long support for people with all health problems. The role of GPs includes in particular the treatment and care of patients in their social context, the coordination and integration function in collaboration with other specialists, and the guidance of the patient in primary and secondary healthcare system. Although only a small proportion of patients in Germany are enrolled and treated in integrated care systems (in 2011, e.g., about 2 million patients were enrolled in integrated care systems [31, 32]), integrated care projects are nonetheless quite important for Germany’s healthcare system: according to the top managers of Germany’s statutory health insurers, integrated care projects serve both (1) as a kind of “laboratory” in which new care approaches are tested and afterwards – in case of success – transferred into standard care, and (2) as a complement to standard care in specific populations or indications [31]. An integrated care approach in Germany with a resident physician as key counsellor and guide for the coordination of care across all health service sectors, is the population-based ‘Integrierte Versorgung Gesundes Kinzigtal’ (‘Healthy Kinzig Valley Integrated Care’—‘GK’) in southwestern Germany: GK members select a primary care partner – typically their family doctor – as their ‘doctor of trust’ for their healthcare. GK is considered a best-practice example of integrated care in Germany [33]. Health promotion and preventive strategies are particularly high priorities in GK [34–37]. For this reason, it can be assumed that - in line with the basic idea of cross-sectoral integrated healthcare - work-related healthcare for working patients could also be better achieved among the practitioners cooperating within GK than in conventional healthcare.

The aim of the study is to determine the proportion of employed GK members aged between 19 and 65 years who have already discussed their future work ability with their family doctor and to present explanatory (independent) variables for the outcome ‘discussion of future work ability with the GP’.

## Methods

### Study design and population

At the time of the survey, about 33,000 people out of around 70,000 residents of the region, had the opportunity to take part in the GK [38]. A random selection of registered GK members is regularly and comprehensively surveyed about their satisfaction with their healthcare [38].

As part of a trend study [38] – i.e. a sequence of cross-sectional studies with the same research question and target group - 3218 randomly selected GK members were surveyed in summer 2017 on their satisfaction with the integrated healthcare system and with their chosen ‘doctor of trust’ (i.e. their family doctor). After the selected members had received the standardized questionnaire by mail in June 2017, they received a written reminder of the survey three weeks later. In addition to questions on satisfaction with their ‘doctor of trust’ and the healthcare system [38], the 2017 survey included for the first time questions on work ability and employment. The trend study was approved by the ethics committee of the University of Freiburg (Az. 294/12_140826). For the purposes of this study, all respondents who indicated to be in employment and who were not older than 65 years were included.

### Elements of the standardized questionnaire

The survey instrument was a standardized questionnaire consisting of 75 individual items.

Additional File 1 provides an overview of the target variables and instruments used in the survey. The questionnaire comprised standardized instruments as well as individual items that were either part of previous surveys or newly added work-related items. Patients’ satisfaction with various aspects of the care provided by their ‘doctor of trust’ as well as their overall satisfaction with this ‘doctor of trust’ were assessed using the questionnaire ‘Weisse-Liste-Ärzte’ [39, 40]. The overall satisfaction with the ‘doctor of trust’ – i.e. their family doctor who, as a rule, is a GP – was reflected in the willingness to recommend this practitioner to their best friend (“Would you recommend this doctor to your best friend?”; response options: definitely not, probably not, maybe, probably, definitely). The survey instrument also included the five items of the EQ-5D (three-level version with response options ‘no’, ‘some’, ‘extreme problems/unable to’) for assessing health-related quality of life and the associated vertical visual analogue scale (EQ-VAS; ‘0’ worst imaginable health status to ‘100’ best imaginable health status) for the assessment of the subjective health status on the survey day [41].

If respondents were employed, they were asked to answer five more questions (see Table 2). Four of these items were directly taken from the German version of the Work Ability Index questionnaire (WAI) [43], i.e. the two indicators WAI-2 (subscale “Work ability in relation to the demands of the job”; 2 items) belonging to the factor ”subjectively estimated work ability and resources” [44, 45] and WAI-6 (subscale “Own prognosis of work ability two years from now”; 1 item) as both show a particularly high predictive power with regard to disability pension [10, 11]. In addition, the categorical description of work-related physical or mental demands was used as first item in this part of the questionnaire (if the work ability index is calculated, this item usually serves as weighting factor for WAI-2). Finally, respondents were asked whether they had ever discussed their assessment of future work ability with their GP (self-developed item; response options: yes/ no).

As the WAI is usually applied only to people in working life, the following information was added below item WAI-6: “Should you retire in two years, please estimate whether you would still be able to carry out your current work - based on your current state of health - at that time.” It was known from previous surveys that the average age of the GK members would be very high. The notice was inserted to avoid missing values among 63–65 year old members and also to record hypothetical future work ability, as the impact of the assessment of (future) work ability on life in retirement is considered influential [12].

### Statistical analysis

All analyses are based on pseudonymized cross-sectional data. All analyses were performed with IBM Statistics SPSS for Windows, version 25 (IBM Corp., Armonk, NY, USA). First, the data was analyzed exploratively in order to identify possible logistic regressions between survey variables and the dependent variable ‘discussion of future work ability with the GP’. This analysis included all respondents who were employed and were aged 65 or younger at the date of survey according to the current retirement age in Germany. Explanatory variables for the target variable ‘discussion of future work ability with the GP’ were analyzed by means of simple (univariate) and multiple logistic regression. To have an objective criterion for exclusion of variables for the sake of stable models we choose in an ad hoc manner a frequency of at least *n* = 25 for each category as being necessary in order to be considered an explanatory variable in logistic regression analysis. To select the explanatory (independent) variables for the multivariate logistic regression model, the regression coefficients of univariate logistic regressions were considered initially. In the multivariate model, variables were tested that were theoretically justifiable and had shown a significant bivariate relationship (*p* < 0.05) with the target variable ‘discussion of future work ability with the GP’. In the case of theoretically similar, highly inter-correlated independent variables, those that made a higher contribution to the explanation of the outcome were selected for the final multivariate model. These variables were checked and selected on the basis of a multiple logistic regression analysis using the selection method “stepwise backward”. The selected variables were used to calculate the final model with the method “enter”. Regression coefficients, odds ratios with the respective two-sided 95% confidence intervals and *p*-values are provided for this model.

## Results

### Response rate, socio-demographic and health-related data

A total of 1168 questionnaires of the third member survey of GK integrated healthcare were evaluated (absolute response rate: 36.6%; evaluable response rate: 36.3%). A comparison between the survey participants and the contacted members who did not participate in the survey did not reveal any significant difference between genders. With regard to age, however, non-participants were on average 56.9 years old, whereas participants were on average 62.3 years old (*p* < 0.001). Out of the survey sample, 475 respondents (40.7%) were maximally 65 years old and stated that they were currently employed. Socio-demographic data are shown in Table 1.

**Table 1:** Sociodemographic data on employed respondents up to 65 years of age ( n = 475)

Sociodemographic Characteristics		
Age (years) *n* = 475	Mean (SD) Median Range	49.6 (10.7) 53 19–65
Gender n = 475	Female	55.6%
	Male	44.4%
Education level *n* = 470	No school leaving certificate	0.6%
	Secondary school certificate	47.2%
	Intermediate maturity	36.8%
	Polytechnic secondary school	1.3%
	Advanced technical college certificate	6.6%
	Abitur (a-level)	7.4%

About half of the respondents (*n* = 237; 49.9%) stated that they did not suffer from any chronic disease; 43.2% (*n* = 205) indicated they suffered from at least one chronic disease, and 6.5% (*n* = 31) indicated they did not know, two respondents (0.4%) did not answer this question.

The mean value of the EQ-5D index (health-related quality of life) was 0.90 (SD = 0.14; range: 0.18–1.0) for 467 respondents with valid scores. When asked about their current state of health on a scale between 0 and 100 (EQ-VAS), the mean value of the 458 valid answers was 77.4 (SD = 15.8; range: 20–100). Whether the respondents would recommend their GP to their best friend was answered by *n* = 5 (1.1%) with ‘definitely not’, *n* = 14 (2.9%) with ‘probably not’, *n* = 67 (14.1%) with ‘maybe’, *n* = 142 (29.9%) with ‘probably’ and *n* = 243 (51.2%) with ‘definitely’; *n* = 4 (0.8%) made no statement.

Table 2 shows the results with regard to the subjective assessment of work-related demands, work ability in relation to these demands (WAI-2), own prognosis of work ability two years from now (WAI-6), and discussion of future work ability with the GP.

**Table 2:** Subjective assessment of work-related demands, work ability and discussion of future work ability with general practitioner (*n* = 475; employed, ≤ 65 years of age)

Characteristic		Number	Percent
Predominant work-related demands^a^ *n* = 473	Mental	126	26.5%
	Physical	88	18.6%
	Both, mental and physical	259	54.8%
Work ability in relation to the physical demands of the job^b^ *n* = 473	Very good	124	26.2%
	Fairly good	225	47.6%
	Average	104	22.0%
	Fairly poor	19	4.0%
	Very poor	1	0.2%
Work ability in relation to the mental demands of the job^c^ *n* = 475	Very good	121	25.5%
	Fairly good	228	48.0%
	Average	102	21.5%
	Fairly poor	22	4.6%
	Very poor	2	0.4%
Own prognosis of work ability two years from now^d^ *n* = 474	It is unlikely	17	3.6%
	I am not sure	105	22.2%
	I am quite sure	352	74.3%
Discussion of future work ability with the general practitioner^e^ *n* = 475	No	389	81.9%
	Yes	80	16.8%
	Missing	6	1.3%

^a^ „Are the demands of your work mainly … - mental work; physical work; an equal amount of mental and physical work?” (WAI, introductory question)

^b^ „How would you evaluate your current work ability in terms of the physical demands of your work?” (WAI-2, item 1)

^c^ „How would you evaluate your current work ability in terms of the psychological demands of your work?” (WAI-2, item 2)

^d^ „In terms of your health, do you feel that you will be able to work in your current profession two years from now?” (WAI-6)

Translations of items 1–4 given according to Work Ability Index™ (updated 2 January 2012, Hopsu L, Lerssi-Uskelin J, Seitsamo J & Tuominen E) [42]

^e^ „Have you ever discussed your assessment of future work ability with your general practitioner?“

### Exploratory data analysis to prepare for multivariate logistic regression

As outlined in the section “Methods”, variables were identified that were related to the discussion of future work ability with the GP in bivariate analysis; for this purpose, simple logistic regressions with the target variable (‘discussion of future work ability with the GP’) were first considered. As possible explanatory variables we examined age, gender, duration of school education, subjective work ability in relation to the physical and mental demands of the job (WAI-2), overall satisfaction with the GP (willingness to recommend him/her to the best friend), and information on chronic diseases (dichotomous: no/don’t know - yes), health-related quality of life (EQ-5D index) and current state of health (EQ-VAS). The results of the univariate regression analyses of the explanatory variables and the target variable are shown in Table 3.

**Table 3:** Explanatory variables for the target variable ‘discussion of future work ability with the general practitioner (GP)’ (no discussion = 0; discussion = 1) - logistic regression analysis – simple (univariate) logistic regression

	Regression-coefficient B	Odds Ratio (OR)	95% confidence interval for OR		*p*-value
			Lower limit	Upper limit	
Work ability in relation to the physical demands of the job^a^	−0.587	0.56	0.41	0.75	< 0.001
Work ability in relation to the mental demands of the job^b^	−0.431	0.65	0.49	0.86	0.003
Satisfaction with GP^c^	0.366	1.44	1.05	1.98	0.023
Chronic disease^d^	0.947	2.58	1.57	4.23	< 0.001
EQ-5D index^e^	−2.672	0.07	0.02	0.29	< 0.001
EQ-VAS^f^	−0.025	0.98	0.96	0.99	0.001
Age	0.007	1.01	0.98	1.03	0.551
Gender^g^	−0.093	0.91	0.56	1.48	0.707
Duration of school education	−0.230	0.80	0.63	1.01	0.059

^a^ very poor = 1; rather poor = 2; moderate = 3; rather good = 4; very good = 5

^b^ very poor = 1; rather poor = 2; moderate = 3; rather good = 4; very good = 5

^c^ Recommendation: definitely not = 1; probably not = 2; maybe = 3; probably = 4; definitely = 5

^d^ no/do not know = 0; yes = 1

^e^ range: 0.205–1

^f^ worst imaginable health state = 0 to best imaginable health state = 100

^g^ male = 0; female = 1

The results of Table 3 may be described in plain words as following: The odds ratio (OR) of a discussion of respondents’ future work ability with their GP is increased (a) the more negatively the respondents assess their current work ability in terms of physical or mental demands of the job, (b) the more satisfied they are with their GP, (c) when they are chronically ill and (d) the more negatively they assess their health-related quality of life or their current state of health, respectively. The sociodemographic variables age, gender and duration of school education are not significantly associated with the target variable ‘discussion of future work ability with the GP’.

### Collinearity analysis

The collinearities of the potential explanatory variables were also taken into account. The correlation analyses (Pearson’s r) showed: Work ability in relation to the physical demands of the job correlated with work ability in relation to the mental demands of the job with r = 0.69; *p* < 0.001 (*n* = 473). EQ-5D index correlated with EQ-VAS with r = 0.58; p < 0.001 (*n* = 453). Variables with higher explanation of variance for the target variable in the multivariate model were work ability in relation to the physical demands of the job (compared to work ability in relation to the mental demands of the job) and current state of health (EQ-VAS) (compared to EQ-5D index).

### Multivariate logistic regression analysis

To select the explanatory variables for the final multivariate model, the significant independent variables that resulted from univariate logistic regressions were subjected to a multiple regression analysis using the selection method “stepwise backward”. Eventually three explanatory variables remained in the model: ‘work ability in relation to the physical demands of the job’, ‘satisfaction with the GP’, and ‘chronic disease’. As a U-shaped relationship between satisfaction with the GP and the discussion of work ability had been found, ‘satisfaction with the GP’ was expressed via two transformed variables (squared and linear). The linear term was obtained by standardizing the variable; the quadratic term was computed as the square of the standardized variable. Table 4 shows the results of the final multivariate logistic regression analysis with these three explanatory variables using the selection method ”enter”).

**Table 4:** Explanatory variables for the target variable ‘discussion of future work ability with the general practitioner (GP)’ – Final multivariate logistic regression analysis (enter; *n* = 462)

	Regression-coefficient B	Odds Ratio (OR)	95% confidence interval for OR		*p*-value
			Lower limit	Upper limit	
Satisfaction with GP (squared)	0.314	1.37	1.14	1.65	0.001
Satisfaction with GP (linear)	0.734	2.08	1.42	3.06	< 0.001
Work ability in relation to the physical demands of the job	−0.558	0.57	0.42	0.79	0.001
Chronic disease	0.607	1.84	1.08	3.13	0.026

Nagelkerke’s pseudo-R^2^ for this model is 0.13. This value can range from 0 to 1. The significant *p*-values of the explanatory variables show the contribution to the target variable. The odds ratio for discussing future work ability with the GP is increased the more the GP would be recommended to the best friend, the worse the current work ability is assessed in relation to the physical demands of the job, and when the respondent suffers from one or more chronic diseases.

## Discussion

### Discussion of future work ability with the GP

Overall, future work ability of GK members of working age was rarely discussed in general practitioner consultations, despite the strong relationship to the GP and GK’s special focus on prevention and health promotion: Only 80 (16.8%) of the employed respondents up to the age of 65 years stated that they had already discussed their appraisal of future work ability with their GP.

In our exploratory approach, the multivariate logistic regression analysis showed significant independent contributions of the variables ‘satisfaction with the general practitioner’ (in the survey assessed as willingness to recommend the ‘doctor of trust’ to one’s best friend), ‘work ability in relation to the physical demands of the job’ and ‘chronic disease’ to a statistical explanation of the target variable ‘discussion of future work ability with the GP’. However, the predictive power of the corresponding logistic regression model (Nagelkerke’s pseudo-R^2^) is rather low (13%). As the present study is a secondary analysis of the GK members’ satisfaction survey, there may be other factors that were not included in the questionnaire but may influence the indication of the discussion of future work ability with the GP within the survey. In addition, we cannot say whether the issue of future work ability was raised by the patient or by the GP in those cases where the topic was discussed.

On the other hand, it is also possible that the discussion with the GP on the issue of future work ability takes place or is remembered very randomly and therefore no further explanatory variables can be found to represent the real practice. Yet, due to the explorative approach of our study and based on the data derived from the patients’ satisfaction survey, this question cannot be answered by the present analysis.

Our sample of GK members, for whom the GP is ‘counsellor’ and ‘guide’ and thus has an overview of the whole healthcare situation of the patient, suggests, however, that a discussion of future work ability of our survey participants with another provider (e.g. medical specialist) did not take place either. This leads us to the assumption that in other community-based care settings there is probably even less discussion about future work ability with the GP.

### Patients’ own prognosis of work ability two years from now

There was a significant difference between those respondents who assessed their future work ability in two years as unlikely or uncertain (negative) and those who had a positive assessment of their future work ability: 12.6% of respondents with a positive assessment of their future work ability in two years had discussed this issue with their GP (*n* = 44 out of *n* = 349), whereas 30.3% of the respondents with a negative self-assessment of their own future work ability had discussed it with their GP (*n* = 36 out of *n* = 119). This higher percentage should be seen as generally positive as the self-assessment of future work ability has a high predictive value for disability pension [5]. A weak to moderate association between one’s own prognosis of future work ability and the discussion with the GP could also indicate that the issue of work ability becomes more relevant with increasing stress and strain. Yet, we had to exclude the question of one’s own prognosis of work ability in two years from the regression analysis due to a possible reverse causality.

### Integration of occupational aspects in general practice

To our knowledge, this is the first work that explores this research question in the context of the German health care system. In our PubMed search with the terms ‘general practitioner’ (MeSH terms or “general practi*”) and ‘work ability’, we obtained no matching article among the 70 hits.

In our survey, the reasons for or against discussing future work ability with the GP were not collected. For further research on this issue, patients’ reasons should be examined as well as the experiences and attitudes of the GP. If patients had already discussed their future work ability with their GP, it would be interesting to know whether those patients came up with the issue themselves or whether it was raised by the GP. With regard to the experiences and attitudes of GPs towards the issue of future work ability of their patients it would be interesting to know how often and why they addressed this topic and what hindering and fostering factors for discussing this issue they described. Various interventions have already been developed and tested in order to draw the attention of both, the working population and GPs, to possible causes of work-related health problems. Training GPs in a group session to better address work-related health problems could not lead to significantly more frequent work-related diagnoses in a cluster-randomized study in the Netherlands [46]: It is plausible, however, that raising the GPs’ awareness of work-related health problems could lead to more frequent work-related diagnoses if the intervention was tailored to the needs of the GPs [46]. A study on the work of physical therapists and the integration of occupational factors of their patients into treatment concludes that a systematic approach, e.g. using questionnaires and guidelines to draw attention to work-related health problems and gain insight into the work of patients, could increase the effectiveness of treatments [47].

To improve healthcare for the employed, there is a need for an increased and closer cooperation between GPs, occupational health physicians or nurses, and employers: information on the health status and working environment of patients could be used to make appropriate modifications at the workplace that would enable sick workers to continue working or return to work more quickly after a period of sickness absence [48]. In addition, employees with early work-related complaints may benefit from preventive measures designed to meet their specific needs such as the respective program of the German statutory pension scheme to protect employability [49] or its successor, the preventive intervention “RV-fit” which are open to all employees who take part in the statutory pension scheme [50]. GPs could support the application of their patients for these specific preventive measures by issuing the necessary medical certificates. On top, they could actively support the utilization of work-related rehabilitation e.g. in case of chronic illness or could help to ensure a graded return-to-work after long sick leave which has been shown to reduce the need of disability pension [51]. Yet, as described above, the awareness of GPs for work-related health problems is often limited [19, 20].

### Relevance of preserving employees’ work ability

The relevance of the issue ‘work ability’ has steadily increased due to demographic changes, the growing demands in working life and the necessity of a longer working time of older employees [1, 52]. The most general retirement age in the EU Member States is 65 years, but in Germany and several other countries the retirement age is being raised to 67 years, whereby Britain and Ireland are even aiming for a retirement age of 68 years [53]. In Germany, the participation of older workers in the labor market tends to increase strongly [54]. Chronic morbidity is strongly age-related [55]. The employment rates, especially in older ages, are essentially lower, partly as a result of the state of health; reduced opportunities for older workers to stay in employment and reduced motivation to remain at work may also play a role [55].

From a societal point of view, the decline in employees’ work ability and/or early retirement leads to high costs for employers, whereas from an individual point of view a good working life promotes the health and work ability of older employees [56]. Workplaces can also be beneficial to the health of their employees and encourage them to stay longer in employment [56]. Employers are also increasingly obliged to take adequate care of the health of their employees, especially of their older workers [1]. It was found that work ability is a strong predictor of early retirement [12, 57] and is also highly related to thinking about retiring earlier [58]. A scoping review recommends that occupational health interventions should be grounded in the promotion of employees’ work ability and should already be implemented for employees younger than 55 years of age [56]. It is a useful strategy focusing on the work ability of older workers in order to maintain and promote their work motivation and health [59, 60].

Against this background, it could be beneficial if GPs and patients had the preservation of the patients’ work ability as a common goal.

### Limitations

Data were collected at a single point of time. Due to the cross-sectional design of the study the odds ratio can be obtained, but no conclusions on causal relationships can be made. Furthermore there might be the possibility of “reverse causality”, i.e. that the perceived future work ability might have influence on the explanatory variables. However, we are rather sure, that this fallacy is not very probable in our analysis. The response rate of 36% might be regarded as moderate or low, but it lies within the range of what may be expected from a postal survey without special incentives for respondents to participate [61–64]. Nonetheless we should consider a possible response bias of our study: it is possible that people who had an increased interest in their personal health were overrepresented in our sample.

## Conclusion

Even in the integrated healthcare system ‘Gesundes Kinzigtal’, a best-practice example of integrated healthcare in which the general practitioner is the patient’s ‘guide’ for the entire healthcare, the employed survey participants (with a median age of 53 years) rarely discussed their assessment of future work ability with the GP. This finding suggests that the issue of future work ability is even less frequently discussed in other community-based care settings. It seems that providers in acute care only sporadically address this issue - despite the great importance of maintaining work ability.

## Supplementary Information

**Additional file 1.**

## Data Availability

The dataset used and analyzed during the current study is available from the corresponding author on reasonable request.

## Abbreviations

EQ-5D: EuroQol five-dimension scale
EQ-VAS: EuroQol visual analogue scale
GK: ‘Integrierte Versorgung Gesundes Kinzigtal’ (‘Healthy Kinzig Valley Integrated Care')
GP: General Practitioner
OR: odds ratio
WAI: Work Ability Index
