# Supplementary material for: Addressing future work ability of employees in GP consultations: results of a cross-sectional study
Source: J Occup Med Toxicol. 2021 Mar 22;16:10. doi: 10.1186/s12995-021-00299-y (PMC7983286; doi:10.1186/s12995-021-00299-y)
Supplement: Supplementary file 1 — Additional file 1. [file 12995_2021_299_MOESM1_ESM.docx]

Additional file 1: Overview of target variables and survey instruments in the questionnaire consisting of 75 items

Table 1: Overview of target variables and survey instruments in the questionnaire consisting of 75 items

| **Target variables** | **Instruments** |
| --- | --- |
| Socio-demographic characteristics | In the questionnaire, no instrument was used; instead, a code had been printed on the questionnaire indicating age, gender and year of enrolment into GK.  Self-developed items for the highest school education, chronic disease(s), and self-reported health status. |
| Patient satisfaction with various aspects of care provided by the ‘doctor of trust’; overall satisfaction with the ‘doctor of trust’ | ‘Weisse-Liste-Ärzte’ questionnaire [1, 2] |
| Satisfaction with GK integrated healthcare   - Patients’ evaluation of the overall quality of health care since enrolment in GK - Patients’ perceived gains in health-related knowledge since enrolment in GK - Patients’ perceived changes in health behaviour since enrolment in GK - Conclusion of health goal agreement with ‘doctor of trust’ - Willingness to re-enrollment - Willingness to recommend GK membership to friends or relatives | Self-developed items [3] |
| Continuity of Care | Subscale of the Nijmegen Continuity Questionnaire (NCQ) and one additional item [4] |
| Health-related quality of life | EQ-5D (three-level version) [5, 6] |
| Subjective health status | EQ-VAS (visual analogue scale) [5, 6] |
| Patient enablement | Self-developed instrument [7] |
| Work ability | 4 items from of the Work Ability Index (WAI) [8] and two self-developed items |

References

1. Bertelsmann Stiftung. [Questionnaire Weisse-Liste-Ärzte: general practitioners and specialists] Fragebogen Weisse-Liste-Ärzte: Hausärzte und Fachärzte: Bertelsmann Stiftung; 2010.

2. Bertelsmann Stiftung. [Weisse-Liste-Ärzte Method documentation: Development of a survey instrument for GP and specialist care] Weisse-Liste-Ärzte Methodendokumentation: Entwicklung eines Befragungsinstruments für die haus- und fachärztliche Versorgung: Bertelsmann Stiftung; 2010.

3. Siegel A, Niebling W. [Individual patient benefit in "Gesundes Kinzigtal" - interim results of a trend study]Individueller Patientennutzen im „Gesunden Kinzigtal“ – Zwischenergebnisse einer Trendstudie. Z Evid Fortbild Qual Gesundhwes. 2018;130:35–41. doi:10.1016/j.zefq.2017.12.003.

4. Uijen AA, Schellevis FG, van den Bosch, Wil JHM, Mokkink HGA, van Weel C, Schers HJ. Nijmegen Continuity Questionnaire: development and testing of a questionnaire that measures continuity of care. Journal of clinical epidemiology. 2011;64:1391–9.

5. EuroQol Group. EuroQol-a new facility for the measurement of health-related quality of life. Health policy. 1990;16:199–208.

6. Moock J. [Preference-based quality of life measurement: The EQ-5D questionnaire] Präferenzbasierte Lebensqualitätsmessung: Der EQ-5D Fragebogen. Physikalische Medizin, Rehabilitationsmedizin, Kurortmedizin. 2008;18:245–9.

7. Siegel A, Ehmann AT, Meyer I, Gröne O, Niebling W, Martus P, Rieger MA. PEN-13: A New Generic 13-Item Questionnaire for Measuring Patient Enablement (German Version). IJERPH. 2019;16:4867. doi:10.3390/ijerph16234867.

8. Tuomi K, Ilmarinen J, Jahkola A, Katajarinne L, Tulkki A. Work ability index. 2nd ed. Helsinki: Finnish Institute of Occupational Health Helsinki; 1998.
